# Energy Loss and Radial Force Variation Caused by Impeller Trimming in a Double-Suction Centrifugal Pump

**DOI:** 10.3390/e23091228

**Published:** 2021-09-18

**Authors:** Qifan Deng, Ji Pei, Wenjie Wang, Bin Lin, Chenying Zhang, Jiantao Zhao

**Affiliations:** 1 Research Center of Fluid Machinery Engineering and Technology, Jiangsu University, Zhenjiang 212013, China; 2112011007@stmail.ujs.edu.cn (Q.D.); 2211911026@stmail.ujs.edu.cn (C.Z.); 2211811011@stmail.ujs.edu.cn (J.Z.); 2 Shenyang Blower Works Group Corporation, Shenyang 110869, China; linbinshengu@hotmail.com

**Keywords:** entropy production, computational fluid dynamics, double-suction centrifugal pump, impeller trimming, radial force

## Abstract

Impeller trimming is an economical method for broadening the range of application of a given pump, but it can destroy operational stability and efficiency. In this study, entropy production theory was utilized to analyze the variation of energy loss caused by impeller trimming based on computational fluid dynamics. Experiments and numerical simulations were conducted to investigate the energy loss and fluid-induced radial forces. The pump’s performance seriously deteriorated after impeller trimming, especially under overload conditions. Energy loss in the volute decreased after trimming under part-load conditions but increased under overload conditions, and this phenomenon made the pump head unable to be accurately predicted by empirical equations. With the help of entropy production theory, high-energy dissipation regions were mainly located in the volute discharge diffuser under overload conditions because of the flow separation and the mixing of the main flow and the stalled fluid. The increased incidence angle at the volute’s tongue after impeller trimming resulted in more serious flow separation and higher energy loss. Furthermore, the radial forces and their fluctuation amplitudes decreased under all the investigated conditions. The horizontal components of the radial forces in all cases were much higher than the vertical components.

## 1. Introduction

Double-suction centrifugal pumps are widely used in water recirculation and water diversion projects, where both a high pump head and a large flowrate are required. Compared with single-suction centrifugal pumps, double-suction centrifugal pumps face more unstable internal flows, caused by the complicated geometry structures of suction chambers and double-suction impellers. The mechanisms of flow instabilities in this type of pump have not been fully demonstrated.

In actual operations, the performance of a given pump needs to be modified to satisfy different requirements for economic reasons, such as reducing the head at the same flowrate. Impeller trimming is an economical approach to the adjustment of pump performance compared with redesigning, changing the rotation speed, and throttling. Furthermore, it is possible to stabilize the inner flows of a double-suction centrifugal pump by trimming the impeller in a specific way [1], since the shortened impeller blades after trimming will change the blade loading, pressure distribution, and other characteristics. However, these inner flows remain indefinite, which means that, conversely, the instability might be made worse. Thus, more attention should be paid to the energy dissipation and operating stability of double-suction centrifugal pumps.

Because impellers are not geometrically similar after impeller trimming, the similarity laws are not fully applicable to the prediction of pump performance in this case. Thus, empirical equations based on statistics are applied to calculate pump performance [1,2]. In many applications, impeller trimming is performed by repeatedly trimming and testing until the pump performance meets the requirements, because inaccurate values might be calculated by the empirical equations [2]. Weme et al. [3] presented a novel prediction method for low-specific-speed pumps to minimize the deviations between predicted and tested pump heads. This method supposed that the deviation between the theoretical (ideal) and tested heads remained unchanged after impeller trimming.

However, this method is not suitable for pumps with a higher specific speed, especially double-suction pumps. Existing research indicates that the gap between the impeller and the volute tongue has a significant impact on the efficiency and radial force of centrifugal pumps [4,5]. Wang et al. [6] reported that trimming impellers in a two-stage self-priming centrifugal pump decreased the radial force on the radial guide vanes and volute; however, the self-priming time increased. Both Yang [7] and Sanjay [8] experimentally investigated centrifugal pumps running in turbine mode and determined that impeller trimming could effectively influence efficiency, but the results varied. Li et al. [9] pointed out that the performance curves of an axial flow fan dropped after impeller trimming, but the efficiency was improved at a large flowrate.

Generally, most studies focused on the hydraulic performance and flow field variation after trimming. However, the changes in energy loss and fluid-induced forces caused by impeller trimming, which are closely connected with efficient and stable operations, were not taken into consideration.

In the past few decades, many researchers have investigated the energy loss in pumps to find a way to improve efficiency. Wang et al. [10] employed an energy loss model and CFD methods to determine the relationship between different loss types and indicated an efficient approach to the optimized design of multistage centrifugal pumps. Shi et al. [11] investigated the energy loss and radial force of a pump running under turbine mode with a gas–liquid two-phase flow.

However, the traditional method could only obtain the rough value of energy loss, and it is still necessary to identify where high energy loss occurs. Thus, the method of loss visualization should be developed. Thermodynamic equations could express hydraulic loss since mechanical energy loss is transformed into heat [12]. According to the second law of thermodynamics, the loss of exergy is entropy production in adiabatic turbomachinery. In other words, the hydraulic loss in hydraulic machinery will eventually transform into thermal energy in the form of entropy production.

In recent years, entropy production theory has been widely used in the estimation of energy loss in hydraulic machinery. Kock and Herwig proposed applying entropy production in CFD based on dissipation in turbulence shear flow [13,14,15]. This enabled the visualization of flow losses in the post process. Böhle et al. [16] adopted this method in a side channel pump and discovered that it was possible to identify high-loss regions in hydraulic machinery. On this basis, Zhang et al. [17] investigated the energy characteristics affected by wrapping angles in a side channel pump. Gu et al. [18] revealed how the clocking position of a vaned diffuser influenced hydraulic loss in a high-power pump and proved the practicability of applying entropy production theory to the analysis of hydraulic loss in stationary domains. Li et al. [19] identified the effects of hydraulic loss characteristics during hysteresis in a pump turbine running in pump mode using entropy production theory. Osman et al. [20] numerically tested different types of channels between two stages of a two-stage double-suction centrifugal pump. Guan et al. [21] used entropy production theory to analyze losses in a double-suction centrifugal pump, and the results indicated that entropy production in the volute greatly impacted the total losses and the scattered wake vortex in the volute increased the hydraulic loss, especially under overload conditions.

On the other hand, impeller trimming not only affects pump performance, but also threatens operational stability. Radial forces change after impeller trimming in different degrees [4]. The radial forces are mainly generated by the nonuniform circumferential distribution of the static pressure at the impeller outlet [1,22]. In centrifugal pumps, the interaction between the blades and the volute tongue worsen the nonuniform pressure distribution due to the asymmetrical structure of the volute. Eventually, these forces act on the surface and lead to rotor vibrations, threatening operational safety [23,24]. Guo and Okamoto [25] experimentally studied the relationship between fluid-induced radial forces and uneven pressure distribution. They also introduced an equation to predict the directions of pressure propagation, radial force whirling, and their dominant frequencies. Tan et al. [26] studied the radial forces inside a centrifugal pump with a vaned diffuser. It was illustrated that, with the decrease of the guide vane outlet angle, the radial forces and their fluctuation amplitudes decreased due to the uniform flow field by the lengthened flow passage. However, the fluctuation frequency and vector distribution did not change. Hao [27] trimmed the blades of a mixed-flow pump for asymmetrical tip clearance. This influenced the radial forces in both magnitude and direction, and the force fluctuation of the asymmetrical tip clearance was six times larger than that of the symmetrical tip clearance. Jiang et al. [28] employed numerical methods to study the influence of the clocking effect on the radial force in a centrifugal pump with a vaned diffuser. The results indicated that, as the guide vane’s trailing edge approached the volute tongue, the pressure fluctuation decreased, but the pump faced larger radial forces and lower efficiency. Based on numerical results, Zou et al. [29] also revealed the relationship between radial forces and vortex structures, and the variation mechanism of radial forces in the startup process when the valve was shut off.

This study analyzed the impacts of impeller trimming on the performance and radial forces of a double-suction centrifugal pump. Firstly, the performance curves of the prototype and the trimming schemes were tested, and the accuracy of the numerical simulation was confirmed. Secondly, the performance reduction and the deviation between the predicted and tested performance curves were explained from the perspective of energy loss, and visualized by entropy production theory along with the relationship with the flow field. Moreover, the variation in the transient radial force was explored.

## 2. Geometric Model and Numerical Simulation

### 2.1. Physical Model of Investigated Pump

The research was carried out on a double-suction centrifugal pump with a specific speed of 24 (by European standards). The basic geometric parameters are given in Table 1. The design flowrate (*Q*_d_) was 500 m^3^/h, and the rotating speed of the shaft (*n*) was 1480 r/min. The manufacturer conducted trimming several times to satisfy the performance demand and found that the performance variation did not follow the empirical equations.

**Figure 1:**
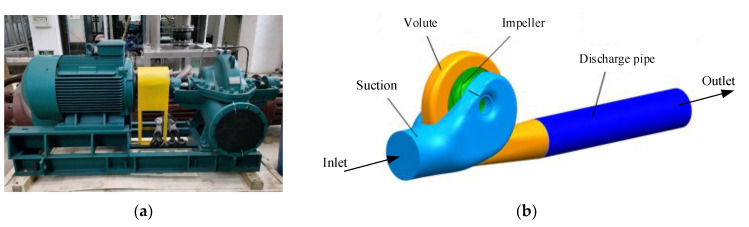
Model pump and three-dimensional model of the flow domain: (**a**) the model pump; (**b**) the 3D flow domain of the model pump.

**Figure 2:**
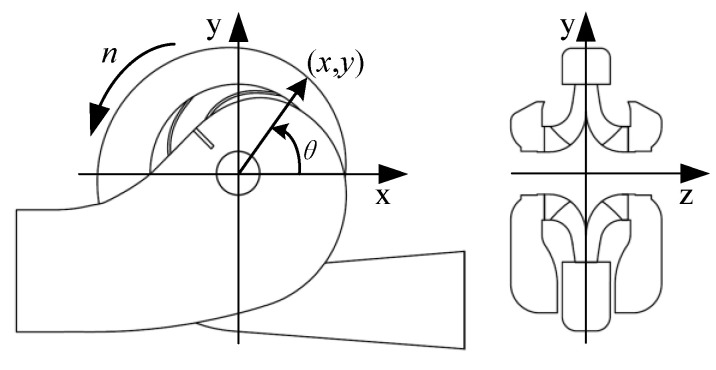
Coordinates of flow domains.

The whole computational fluid domain included a semi-spiral suction chamber, a shrouded double-suction impeller, and a volute casing, as described in Figure 1. A discharge pipe was added after the volute to decrease the effects of the backflow in the volute discharge diffuser in the numerical simulation. For the impeller, there was no center rib between the two blade channels. As shown in Figure 2, the rotating axis of the impeller is *z*, and pump’s symmetry plane is *xy*. The angle between the point (*x*, *y*) and the *x* direction is *θ*, and the angle for the volute tongue is *θ* = −71° (289°).

### 2.2. Trimming Schemes

**Figure 3:**
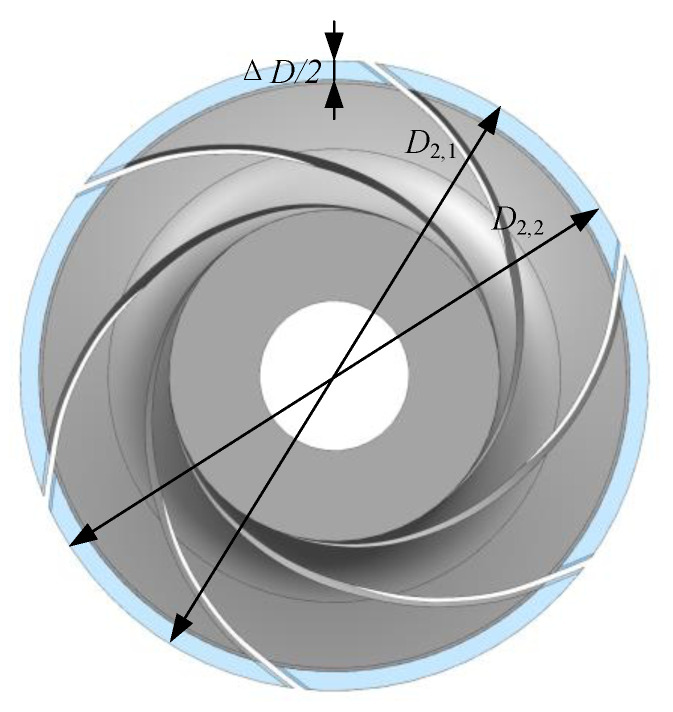
Schematic of the trimming method.

**Figure 4:**
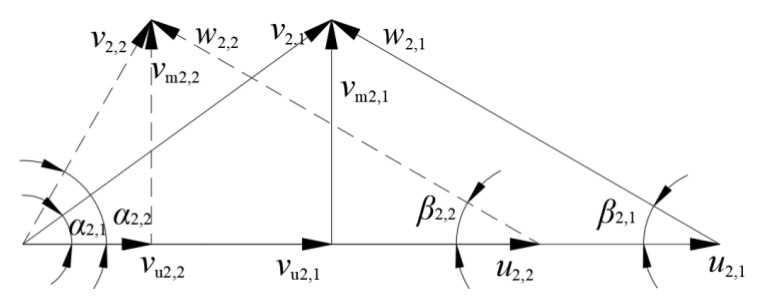
Velocity at blade trailing edge before and after trimming at a given flowrate.

The impeller was trimmed at a constant radius, as shown in Figure 3, where *D*_2,1_ is the original impeller outlet diameter, *D*_2,2_ is the diameter after trimming, and the trimming size is ∆*D*. In this study, the impellers were trimmed by 11 mm, 22 mm, and 33 mm, respectively. The variation of the impeller parameters, including the area and blade angle at outlet section (*A*_2_ and *β*_b2_), are shown in Table 2. Both *A*_2_ and *β*_b2_ remained almost unchanged in Schemes 1 and 2, while the area and blade angle decreased in Scheme 3. Figure 4 shows the velocity at the blade trailing edge at a given flowrate, where the symbols followed by subscript 1 (such as *v*_2,1_) are those before trimming, and those followed by 2 are those after trimming (such as *v*_2,2_).

The circumferential velocity *u*_2_ decreased after trimming due to the smaller blade outlet diameter. The *v*_u2_ value decreased because of a smaller *u*_2_, while α_2_ increased, as shown in the dashed lines in Figure 4. In Schemes 1 and 2, because *β*_b2_ and *A*_2_ remained unchanged, *β*_2_ (relative flow angle) and *v*_m2_ also remained unchanged. In Scheme 3, *β*_2_ decreased while *v*_m2_ increased. Eventually, the relationship between α_2_ in the three schemes was: Scheme 1 < Scheme 2 < Scheme 3.

### 2.3. Mesh Generation and Numerical Setup

**Figure 5:**
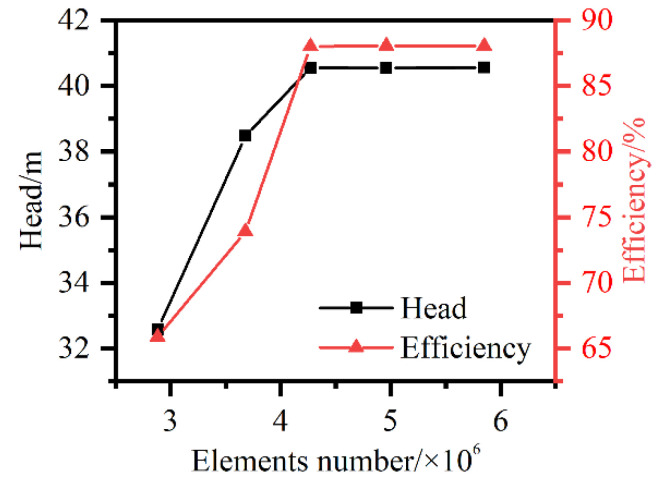
Grid sensitivity based on head and hydraulic efficiency.

**Figure 6:**
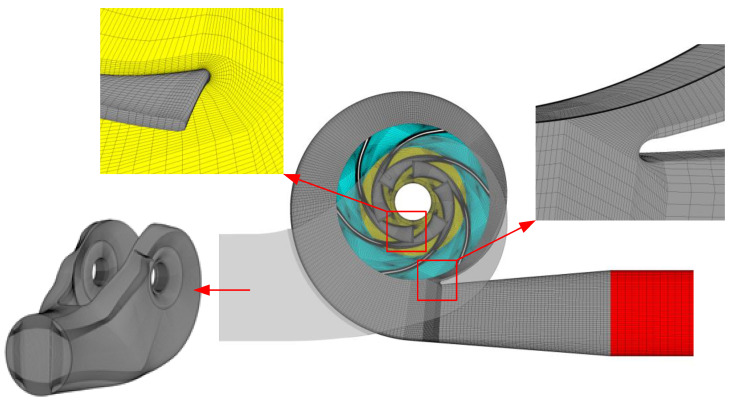
Details of hexahedral grids of flow domains.

ICEM CFD was applied to generate hexahedral grids for all the components. The influence of the element number on the original model was investigated based on the pump head and hydraulic efficiency under the design conditions, as shown in Figure 5. Both the head and hydraulic efficiency curves tended to remain stable when the element number reached 4.96 million. Finally, in terms of the mesh quality and quantity, grids with approximately 5.85 million elements for each scheme were adopted. The grid number, the minimum element angle, and the maximum aspect ratio of each component are listed in Table 3. The *y*^+^ value was lower than 100, which was enough for the RANS model employed here. As shown in Figure 6, the grids were refined near physical walls, especially near the blades and volute tongue.

The commercial software ANSYS CFX was employed, and the SST *k–ω* turbulence model was adopted to solve the RANS equations. The working fluid was water. The boundary conditions were set according to the actual situation. The outlet was mass flow, while the inlet was total pressure, as given in Table 4. Moreover, the inlet’s total pressure was assigned as 1 atm. The interfaces between the rotational and stationary domains were Frozen Rotor under steady state and switched to Transient Rotor Stator in the transient simulation. The high-resolution scheme was applied for advection and other transient terms with the second-order backward Euler scheme. For each case, the RMS residual was 10^−5^. The steady state simulations iterated 500 steps, then the results were utilized to initialize the transient simulations. In the transient simulations, the timestep was the time for rotating 3° (3.38 × 10^−4^ s). The maximum iteration number of each step was five for the sake of time consumption and simulation accuracy. Finally, the results after 13 revolutions (0.527 s) were generated and the last three revolutions were chosen for further analysis. Furthermore, the pressure at the pump inlet and outlet were calculated by the mass-flow average value, and the performance characteristics were calculated by the average value of the last three revolutions.

### 2.4. Hydraulic Loss Estimation Methods

According to the second law of thermodynamics, the specific entropy, *s*, is a state variable that grows in every irreversible process. As RANS equations are solved, the entropy transport equation can be written in the time-averaged version using time-averaged and fluctuation components [15,16], given by Equation (1):

(1) $$\begin{array}{l} \rho \left(\frac{\delta \bar{s}}{\delta t}+{\bar{v}}_{x}\cdot \frac{\delta \bar{s}}{\delta x}+{\bar{v}}_{y}\cdot \frac{\delta \bar{s}}{\delta y}+{\bar{v}}_{z}\cdot \frac{\delta \bar{s}}{\delta z}\right)=\\ -\bar{\mathrm{div}\left(\frac{\vec{q}}{T}\right)}-\rho \left(\frac{\bar{\delta {\bar{v}}_{x}{}^{\prime }s^{\prime }}}{\delta x}+\frac{\bar{\delta v_{y}{}^{\prime }s^{\prime }}}{\delta y}+\frac{\bar{\delta v_{z}{}^{\prime }s^{\prime }}}{\delta z}\right)+\bar{\left(\frac{\Phi_{D}}{T}\right)}+\bar{\left(\frac{\Phi_{\theta }}{T^{2}}\right)} \end{array}$$

where $\bar{\mathrm{div}(\vec{q}/T)}$ is the reversible heat transfer and $\bar{\left(\Phi_{\theta }{/T}^{2}\right)}$ is the irreversible part caused by the heat transfer. As the thermal transmission is negligible here, these two terms can be ignored. The second part on the right is infinitesimal and of a higher order, so it can also be left out. Finally, the remaining item, $\bar{(\Phi_{D}/T)}$, is the irreversible part produced by friction, which results from viscosity and turbulence; this term is dominant for calculating entropy production in hydraulic machinery, as shown in Equation (2).

(2) $$\bar{\left(\frac{\Phi_{D}}{T}\right)}=\frac{\Phi_{\bar{D}}}{\bar{T}}+\frac{\Phi_{D^{\prime }}}{\bar{T}}$$

The first term on the right is the entropy production rate by viscous dissipation, which can be calculated directly in CFD post using Equation (3). The second term is caused by turbulence, as illustrated in Equation (4). Because the velocity fluctuation components, $v_{x}{}^{\prime }$, $v_{y}{}^{\prime },\;$and $v_{z}{}^{\prime }$, cannot be solved by the RANS model, Kock [16] proposed another approach to it by using Equation (5).

(3) $$\begin{array}{l} \Phi_{\bar{D}}=\mu [2\cdot \left[{\left(\frac{\partial {\bar{v}}_{x}}{\partial x}\right)}^{2}+{\left(\frac{\partial {\bar{v}}_{y}}{\partial y}\right)}^{2}+{\left(\frac{\partial {\bar{v}}_{z}}{\partial z}\right)}^{2}\right]\\ +{\left(\frac{\partial {\bar{v}}_{y}}{\partial x}+\frac{\partial {\bar{v}}_{x}}{\partial y}\right)}^{2}+{\left(\frac{\partial {\bar{v}}_{z}}{\partial y}+\frac{\partial {\bar{v}}_{y}}{\partial z}\right)}^{2}+{\left(\frac{\partial {\bar{v}}_{x}}{\partial z}+\frac{\partial {\bar{v}}_{z}}{\partial x}\right)}^{2}] \end{array}$$

(4) $$\begin{array}{l} \Phi_{D^{\prime }}=\mu [2\left[{\left(\frac{\partial v_{x}{}^{\prime }}{\partial x}\right)}^{2}+{\left(\frac{\partial v_{y}{}^{\prime }}{\partial y}\right)}^{2}+{\left(\frac{\partial v_{z}{}^{\prime }}{\partial z}\right)}^{2}\right]\\ +{\left(\frac{\partial v_{y}{}^{\prime }}{\partial x}+\frac{\partial v_{x}{}^{\prime }}{\partial y}\right)}^{2}+{\left(\frac{\partial v_{z}{}^{\prime }}{\partial y}+\frac{\partial v_{y}{}^{\prime }}{\partial z}\right)}^{2}+{\left(\frac{\partial v_{x}{}^{\prime }}{\partial z}+\frac{\partial v_{z}{}^{\prime }}{\partial x}\right)}^{2}] \end{array}$$

(5) $$\Phi_{D^{\prime }}=\rho \varepsilon$$

where *ε* is the dissipation rate of turbulent kinetic energy, which is already available in the turbulence model. To quantify the influence of entropy production, the power lost by entropy production in a certain domain can be estimated by integrating the dissipation rate in the whole domain, as illustrated by Equation (6).

(6) $$P_{\mathrm{EP}}={∭}_{V}\Phi_{D}dV$$

## 3. Experiments and Validation

### 3.1. Experimental Setup

**Figure 7:**
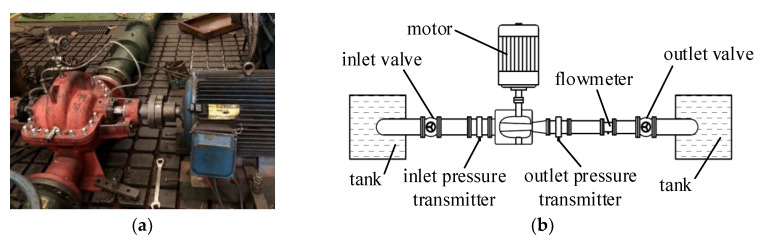
Test rig and test pump: (**a**) the tested pump on the test rig; (**b**) the diagram of the test rig.

The experiments were conducted on an open test rig, schematically shown in Figure 7. In the experiments, an electromagnetic flowmeter was used to measure the flowrate. The pipe upstream of the flowmeter was 2 m, which was long enough to ensure uniform inflow to the flowmeter; the pipe downstream of the flowmeter was 1.5 m, in order to minimize the effect of the valve. The length of the pump suction pipe was also 2 m in order to make the inlet smooth. The data was only collected if all the parameters remained unchanged or changed periodically in order to ensure steady operating conditions and accurate data.

**Figure 8:**
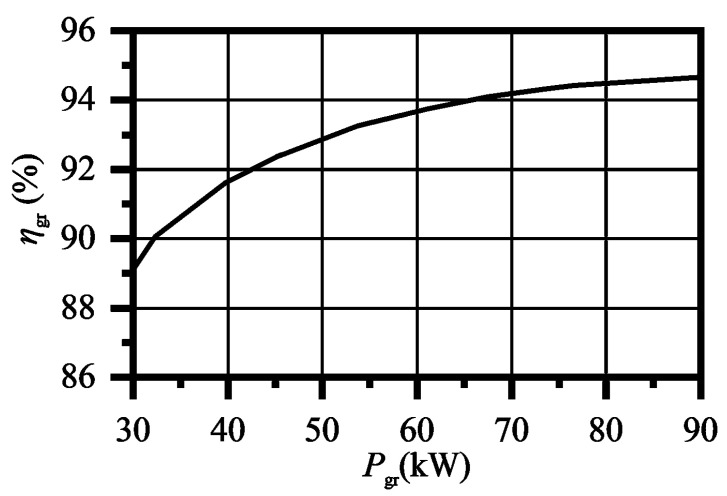
Motor efficiency.

The electric power was measured and converted into shaft power, as shown in Equation (7). In the equation, *η*_motor_ is the motor efficiency, which was measured before the experiments, shown in Figure 8. The voltage and current of the motor are *U* and *I*, respectively, and cos*φ* is the power factor of the motor. The uncertainty of all the instruments was less than 0.25%.

(7) $$P=\eta_{\mathrm{motor}}\sqrt{3}UI\cos\varphi$$

### 3.2. Experimental Results

The experiments were conducted on the prototype and Scheme 1. The performance of Scheme 1 was also predicted by corrected empirical equations for double-suction centrifugal pumps, illustrated by Equation (8) [1,2]

(8) $$\left\{ \begin{array}{l} \frac{H^{\prime }}{H}={\left(\frac{{D^{\prime }}_{2}}{D_{2}}\right)}^{1/0.42}\\ \frac{P^{\prime }}{P}={\left(\frac{{D^{\prime }}_{2}}{D_{2}}\right)}^{3} \end{array}\right.$$

**Figure 9:**
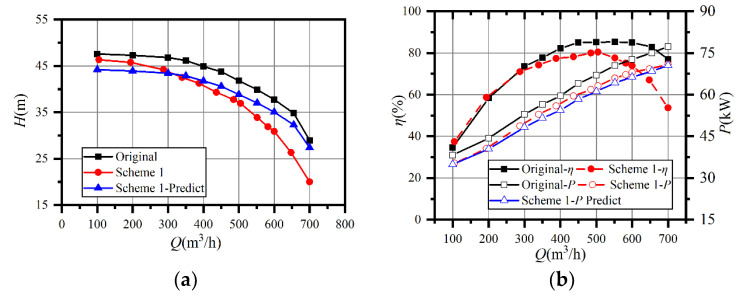
Comparison between test and predicted performance curves with different impellers: (**a**) comparison of head curves; (**b**) comparison of power and efficiency curves.

The tested and predicted performance curves are presented in Figure 9. The predicted head curve differed little from the test value at part-load, and the maximum deviation was 4.6% at 0.2*Q*_d_. The predicted head became higher than the tested value when the flowrate was larger than 0.7*Q*_d_, as the predicted curve decreased more slowly than the test. The deviation between the tested and predicted curves increased with a flowrate of 5.1% at *Q*_d_ and 37.2% at 1.4*Q*_d_. The inverse situation occurred in the power prediction since the deviation between the predicted and actual curves was tiny. Because the head dropped much more than expected, the efficiency decreased dramatically after impeller trimming, and the best efficiency point shifted from 550 m^3^/h to 500 m^3^/h. Meanwhile, the prototype had a flat efficiency curve between 0.7*Q*_d_ and 1.4*Q*_d_, where the efficiency was higher than 78%.

Since the predicted power curve deviated little from the test, it might be imagined that the capacity of energy conversion in the impeller could have been expressed by empirical equations. However, some unpredictable factors can lead to imprecision in head prediction at a large flowrate. In previous studies, the energy loss was considered constant. However, the pump had a lower head at the same flowrate and the volume and disk friction loss decreased after impeller trimming. The enlarged gap resulted in increased flow circulation in the annular region between the blade trailing edge and the volute tongue, which increased hydraulic loss [3]. The variation of hydraulic loss might have led to a difference between the prediction and the experiment.

### 3.3. Numerical Results and Validation

**Figure 10:**
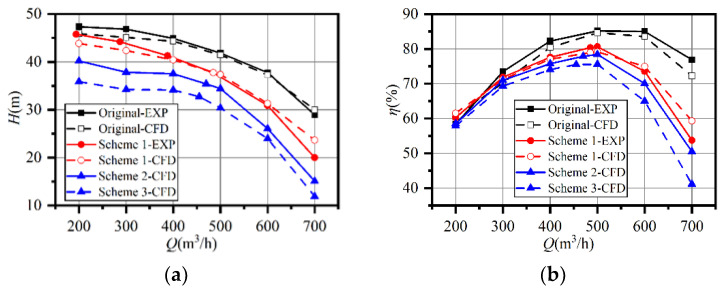
Comparison of experiments and simulation results: (**a**) head curves; (**b**) efficiency curves.

Numerical simulations were conducted on all the schemes, under various operating conditions, from 0.4*Q*_d_ to 1.4*Q*_d_. The experimental and numerical results of the prototype and Scheme 1 were compared. Figure 10 shows the comparison between the experimental and numerical performance characteristics. The maximum deviation in head curves was 7.81% at 1.4*Q*_d_ in Scheme 1. The efficiency in both cases also showed the same trend as in the experiments. The prototype had a flat efficiency curve between 0.8*Q*_d_ and 1.2*Q*_d_, while the curves became steeper after trimming. However, the best efficiency points of Schemes 2 and 3 were still 500 m^3^/h. Thus, the simulation results were reliable and achieved a good correlation with the experiments.

## 4. Results and Discussion

### 4.1. Analysis of Hydraulic Loss

As the hydraulic performance is determined by the total pressure difference, the total pressure loss in rotating and stationary domains is estimated in the form of head drop using Equations (9) and (10) respectively. To reveal the effects of impeller trimming on hydraulic loss in each case, the loss coefficient (∆H*) is defined as the proportion of lost power in impeller input power by using Equation (11).

Stationary domain:

(9) $$\Delta H=\frac{p_{T1}-p_{T2}}{\rho g}$$

Rotational domain:

(10) $$\Delta H=\frac{P_{is}-(p_{T2}-p_{T1})Q}{\rho gQ}$$

(11) $$\Delta H^{*}=\frac{\Delta H\rho gQ}{P_{is}}$$

where *p*_T1_ and *p*_T2_ are the total pressure at the inlet and outlet of each component and *P_is_* is the impeller’s input power, which is calculated by numerical simulation. The efficiency calculated in the simulation is the pump output power divided by the impeller input power, so Equation (11) could be transformed into Equation (12).

(12) $$\Delta H^{*}=\frac{\Delta H}{H}\eta$$

**Figure 11:**
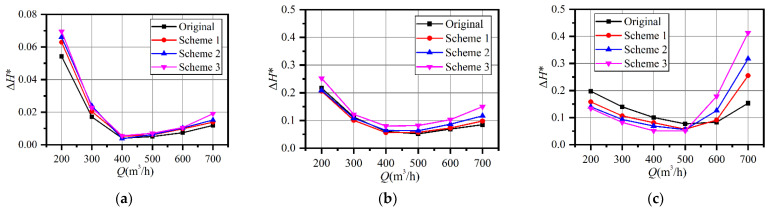
Comparison of the loss coefficient of each component: (**a**) loss coefficient of suction chamber; (**b**) loss coefficient of impeller; (**c**) loss coefficient of volute casing.

Figure 11a indicates that the suction chamber contributed little to the hydraulic loss. The maximum ∆H* appeared at 0.4*Q*_d_, while the minimum value appeared at 0.8*Q*_d_ for all the schemes. The value only increased slightly with the increasing flowrate at overload. The ∆H* increased slightly with increasing trimming size because of the decreasing impeller input power after trimming.

As shown in Figure 11b, the ∆H* of the impeller had the same trend as the suction chamber, but the value was much higher. Clearly, the ∆H* of Scheme 3 was the highest under all conditions. The relationship between the other three schemes was variable since the ∆H* of Scheme 1 was the smallest at part-load. Under the design conditions, the ∆H* of the original impeller was the smallest. The ∆H* increased with the increasing trimming size at overload, and the maximum deviation between the prototype and Scheme 3 was 0.07.

As illustrated in Figure 11c, the situation in the volute was different. When the flowrate was no larger than *Q*_d_, the highest ∆H* appeared in the prototype and the maximum value was 0.20. The ∆H* decreased when the trimming size grew larger. The large deviation between the prototype and Scheme 1 at part-load might be one of the main explanations for the efficiency improvement of Scheme 1, as shown in Figure 10b. However, the ∆H* curves of the trimming schemes were steeper than in the original. The ∆H* of the prototype decreased and became the smallest at 1.2*Q*_d_, although it was the highest at *Q*_d_. The ∆H* of the volute increased with the increasing trimming size, but the deviation was much larger than that of the suction chamber and the impeller. The ∆H* was only 0.083 and 0.090 for the prototype and Scheme 1, respectively, at 1.2*Q*_d_, while the value for Scheme 3 was 0.18. The value increased to 0.15 for the prototype, 0.25 for Scheme 1, and 0.41 for Scheme 3 at 1.4*Q*_d_. To explain this phenomenon, energy dissipation and internal flow are discussed in the next section.

According to Figure 10, the efficiency deviation between the prototype and Scheme 1 is 13.2% and the deviation of their ∆H* was 0.1. It seems the increasing hydraulic loss in the volute contributed the most to the efficiency drop in Scheme 1. The increasing loss in the volute at overload might explain the steep efficiency curves after impeller trimming.

### 4.2. Comparison of Total Pressure Loss and Entropy Production

**Figure 12:**
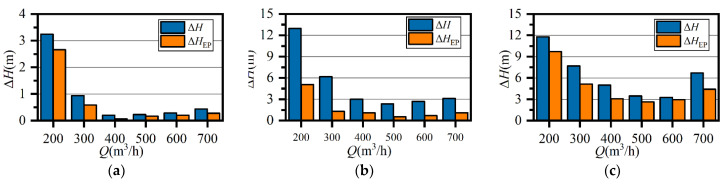
Comparison between total pressure loss and entropy production of the prototype: (**a**) suction chamber; (**b**) impeller; (**c**) volute casing.

The hydraulic losses calculated from the total pressure difference (Equations (9) and (10)) and entropy production (Equation (6)) are compared in Figure 12. The loss calculated by entropy production was transformed by Equation (13) to ensure the same dimension. In stationary domains, entropy production accounted for 72–91% of the total pressure loss. Thus, it was possible to visualize the analysis of flow loss in stationary domains by using entropy production theory. The difference between ∆*H* and ∆*H*_EP_ may have resulted from the high velocity gradient near the wall surface, where a small value of *y*+ is required to capture wall friction [15,21]. Hence, it is reasonable to analyze the losses inside the volute to reveal the mechanism of energy dissipation by using the visualization methods of entropy production, as has been proven in previous studies [18,19,20,21].

(13) $$\Delta H_{EP}=\frac{P_{EP}}{\rho gQ}$$

### 4.3. Analysis of Flow Loss Distribution

To determine where high hydraulic loss occurred and the relationship with the flow pattern, the energy dissipation visualized by entropy production theory and the velocity distribution under different operating conditions inside the volute were studied. Schemes 1 and 2 were compared with the original pump.

**Figure 13:**
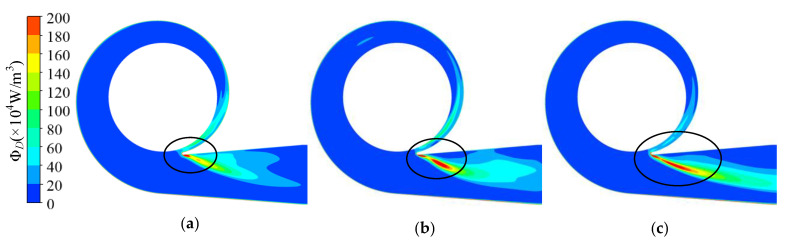
Dissipation distribution on the volute middle section under 1.4*Q*_d_ condition: (**a**) prototype; (**b**) Scheme 1; (**c**) Scheme 2.

**Figure 14:**
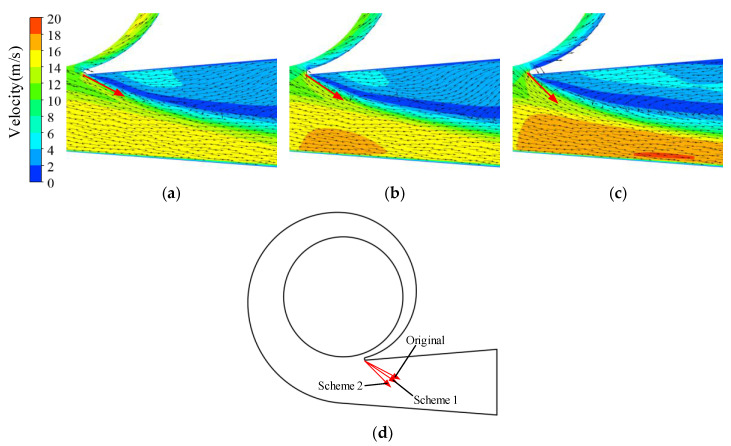
Velocity vector distribution in volute diffuser under 1.4*Q*_d_ condition: (**a**) prototype; (**b**) Scheme 1; (**c**) Scheme 2; (**d**) comparison of velocity directions near the volute tongue.

**Figure 15:**
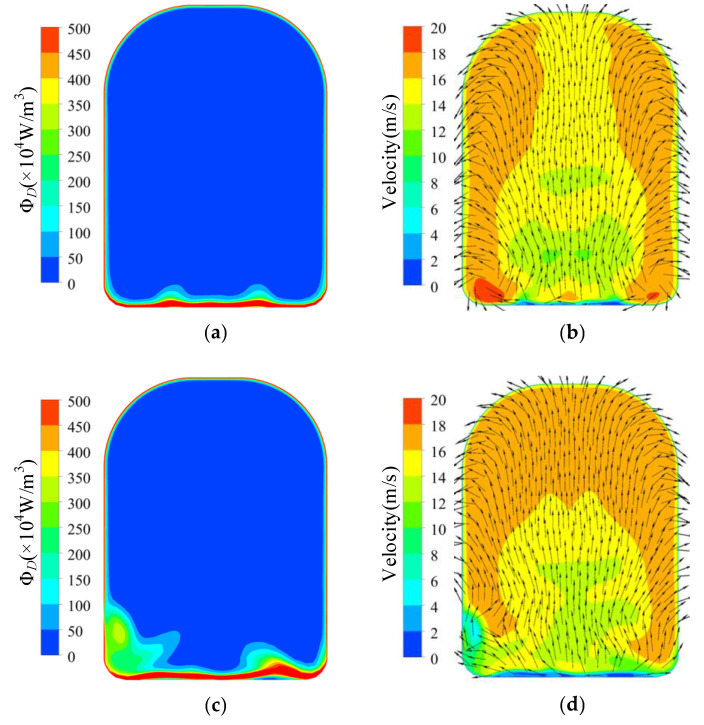

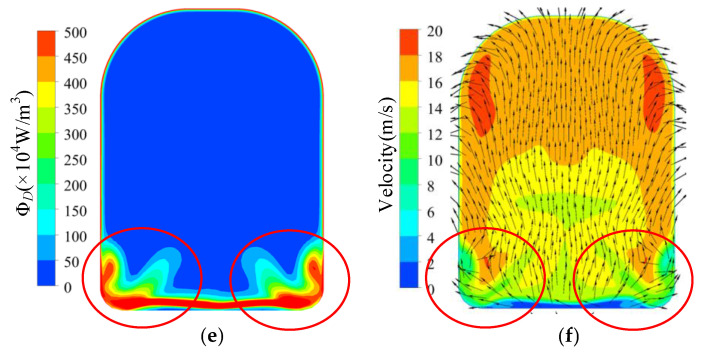
Dissipation and velocity distribution at volute throat under 1.4*Q*_d_ condition: (**a**) dissipation of prototype; (**b**) velocity of prototype; (**c**) dissipation of Scheme 1; (**d**) velocity of Scheme 1; (**e**) dissipation of Scheme 2; (**f**) velocity of Scheme 2.

The energy dissipation distribution in the volute at 1.4*Q*_d_ is illustrated in Figure 13. The energy dissipation in the volute discharge diffuser was much higher than in other areas in the volute. The high dissipation area was in front of the tongue and in the discharge diffuser in all cases and expanded with increasing trimming size. However, the dissipation in the volute decreased in the circumferential range of −71°–0°. According to Figure 14a, there was severe flow separation in the discharge diffuser. The velocity direction near the tongue is shown with red arrows, and a comparison is presented in Figure 14d. As described by Gülich [1], a large incidence angle results in flow separation and the stalled fluid blocks the channel. The fluid accelerates when it goes through the throat area. The incidence angle increases after impeller trimming because of the increasing impeller outlet angle. The flow separation deteriorates after trimming and velocity at the volute throat is higher, as shown in Figure 15. The energy dissipation also increases near the tongue because of the high velocity gradient, which contributes to viscous dissipation, as described in Equation (4). Comparing Figure 13 and Figure 14, it is obvious that the flow separation and the mixing of the main flow and the stalled fluid can explain the high dissipation. In the trimming, the increased incidence angle resulted in a stronger separation flow and a higher velocity gradient. Finally, the energy dissipation in this area was higher than in the original pump, and the efficiency decreased with the increasing trimming size. Although the energy dissipation in the volute (−71° < θ < 0°) decreased after impeller trimming, the reduction in energy loss here was much lower than the increase in the discharge diffuser and had little influence on the efficiency.

**Figure 16:**
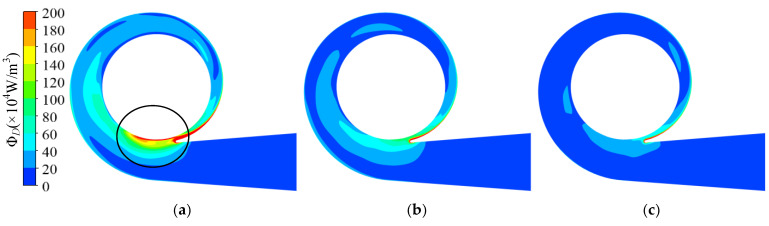
Dissipation distribution on volute middle section under 0.6*Q*_d_ condition: (**a**) prototype; (**b**) Scheme 1; (**c**) Scheme 2.

**Figure 17:**
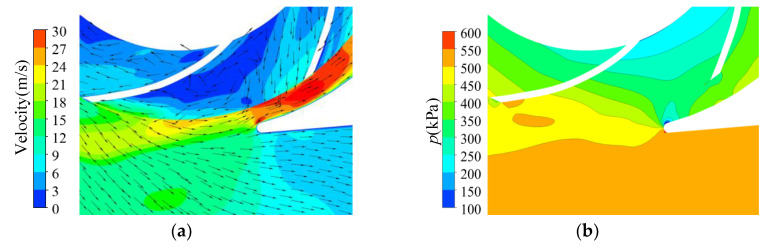
Velocity and pressure distribution near volute tongue in prototype under 0.6*Q*_d_ condition: (**a**) velocity distribution; (**b**) pressure distribution.

The energy dissipation at 0.6*Q*_d_ is shown in Figure 16. In the prototype, the energy dissipation on the inside of the tongue (*θ* = –71°–0°) was much higher than in other areas, which mainly resulted from the high velocity at the blade trailing edge. As illustrated in Figure 17a, some fluid did not enter the discharge diffuser but returned into the spiral section, which increased the velocity in this area. Both the high velocity and the mixing of the backflow and the impeller outflow resulted in high energy dissipation in this area. For the high dissipation area near the volute inlet (circumferential position *θ* = 270°, circled in the picture), the outflow with high velocity from the impeller mixed with the main flow in the volute, and the momentum exchange and high velocity gradient lead to high energy dissipation.

**Figure 18:**
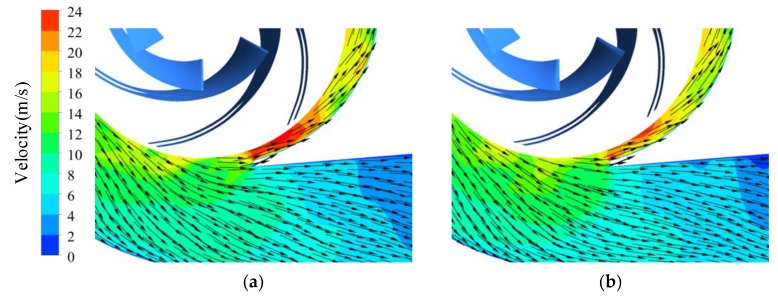
Velocity distribution near volute tongue in trimmed schemes under 0.6*Q*_d_ condition: (**a**) Scheme 1; (**b**) Scheme 2.

As illustrated in Figure 16b,c, the high dissipation area decreased with increasing trimming size. As shown in Figure 18, the trimmed impeller achieved a more uniform flow pattern near the tongue and the velocity on the inside of the tongue decreased conspicuously compared with the prototype because of the decreased blade tip velocity and the enlarged clearance between the blade trailing edge and the volute tongue.

### 4.4. Comparison of Radial Forces

The radial forces acting on the blade surfaces were calculated in the numerical simulation. The forces solved by CFX software were in the relative frame. The value and direction of the forces acting on the impeller’s surface were calculated in the rotating frame, which is inconvenient for analysis in an absolute frame. These forces eventually acted on the bearings and the pump casing, which were in the stationary frame (absolute frame). Thus, the forces were transformed into the stationary frame in the study.

**Figure 19:**
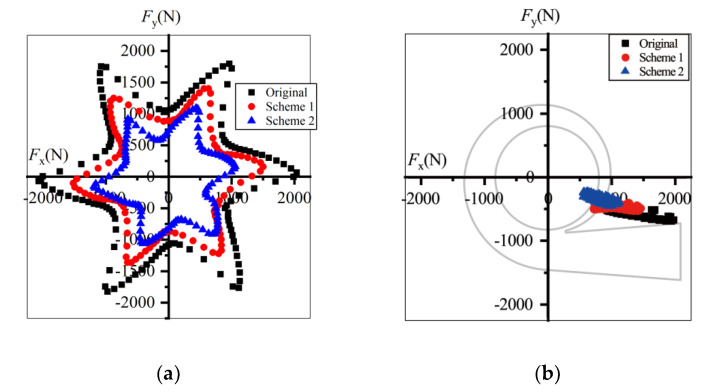
Radial forces under 0.6*Q*_d_ condition: (**a**) in the rotating frame and (**b**) in the stationary frame.

Illustrated in Figure 19 are the radial forces at 0.6*Q*_d_ in one revolution. The forces and their fluctuation amplitudes decreased after trimming. The maximum force of the prototype was 2089.4 N, decreasing to 1199.6 N in Scheme 2, and the peak-to-peak value decreased from 1064.0 N to 599.4 N, almost half that of the prototype. Thus, the bearing loads and shaft deflection decrease after impeller trimming and the operational stability and life of pumps can be extended. In the stationary frame, the forces in all schemes displayed the same direction, pointing to the pump outlet in the fourth quadrant. *F*_x_ was far greater than *F*_y_, so the radial force might have caused the horizontal thrust and vibration.

**Figure 20:**
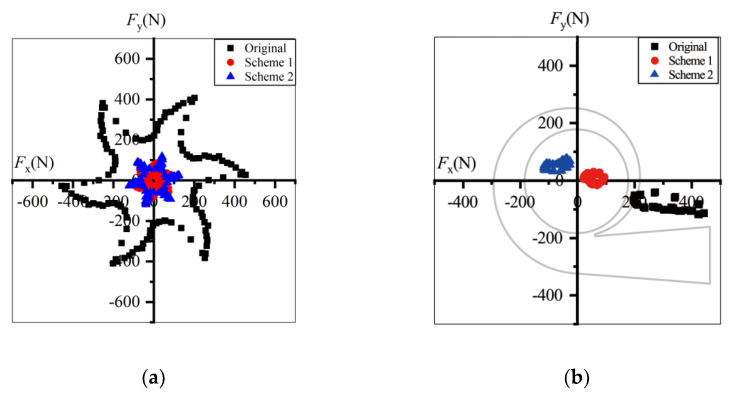
Radial forces under *Q*_d_ condition: (**a**) in the rotating frame (**b**) in the stationary frame.

Under the design conditions, the radial forces decreased after impeller trimming, and Scheme 1 had the smallest radial forces. The maximum force value in Scheme 2 was only one-fifth of that in the prototype. As shown in Figure 20b, the force direction of the prototype did not change compared with that at 0.6*Q*_d_. The direction of *F*_x_ did not change, while *F*_y_ fluctuated around *F*_y_ = 0 in Scheme 1. However, the force direction changed to the second quadrant in Scheme 2.

**Figure 21:**
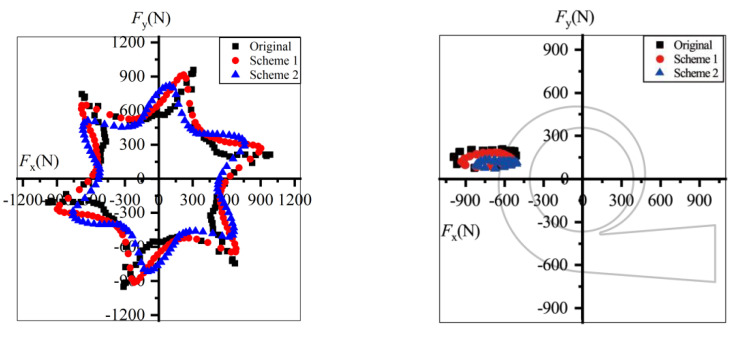
Radial forces under 1.4*Q*_d_ condition: (**a**) in the rotating frame; (**b**) in the stationary frame.

As shown in Figure 21, under 1.4*Q*_d_ conditions the force direction of all the schemes changed to the second quadrant and the value of the radial forces decreased after impeller trimming. Comparing Figure 19 and Figure 20, the deviation between the three schemes was small.

The radial forces at 0.6*Q*_d_ were greater than those under overload conditions. *F*_x_ was far greater than *F*_y_ under all conditions. The horizontal component might have been dominant in terms of flow excitation radial forces. Furthermore, the flow excitation radial forces calculated in Figure 19, Figure 20 and Figure 21 were in the range demonstrated in previously the published data, and the direction of the radial forces was the same as in Gülich [1]. The accuracy of the radial force calculation can therefore be verified.

## 5. Conclusions

This research studied variations in hydraulic loss and radial force after impeller trimming in a double-suction pump. Numerical simulations and experiments were conducted. Entropy production theory was applied to analyze hydraulic dissipation. The radial forces were also compared in both rotating and stationary coordinate systems. The following conclusions were reached:

(1) The empirical equations used to predict pump performance after impeller trimming did not accurately predict the head in the investigated pump. The deviation between the tested and predicted head curves was large at overload. The main reason was the increasing hydraulic loss in the volute after impeller trimming, which is ignored in empirical equations. The predicted power was consistent with the tested values at all the flowrates studied. Thus, the load on the shaft and the capacity of the conserved energy could be predicted after impeller trimming.

(2) The prototype had a wide range of high efficiency (>80%) between 0.8 and 1.2*Q*_d_, but the efficiency dropped in all the trimmed cases. This phenomenon was the result of the increasing loss proportion in the volute after impeller trimming. The hydraulic loss calculated by the total pressure difference and entropy production were in good agreement in the stationary domains.

(3) The high dissipation area was located near the volute tongue and the discharge diffuser due to the flow separation and mixing of the backflow and main flow at overload. The hydraulic loss in these areas increased after trimming because the increased incidence angle on the outside of the tongue strengthened the separation flow and backflow. The hydraulic loss under part-load conditions decreased after trimming because of the reduction in the blade tip velocity. As a result, the empirical equations overestimated the hydraulic loss under part-load conditions, and underestimated it under overload conditions.

(4) The radial forces under the design and part-load conditions decreased more severely after trimming than under overload conditions. Under the design conditions, the force direction changed with the increased trimming, while under other conditions the original direction was maintained. However, the horizontal component of the radial force was greater than the vertical component in all cases, so the pump mainly experienced horizonal impact when operating.

Since the pump displayed a steeper efficiency curve and the effects on the fluid-induced radial force were not distinct at overload, it is acceptable to perform impeller trimming on a given pump if the part-load operation is regular. Furthermore, if trimming is considered in the impeller design, the blade angle should have a decreasing trend when approaching the trailing edge to minimize the increase in the impeller’s outflow angle.

## Figures and Tables

**Table 1:** Geometric parameters of the model pump.

	Parameters	Symbol	Unit	Value
Suction	Inlet diameter	*D*_s_	mm	250
Impeller	Inlet diameter	*D*_1_	mm	192
	Outlet diameter	*D*_2_	mm	365
	Outlet width	*b*_2_	mm	46
	Blade number	*Z*	-	6
	Blade inlet angle	*β*_b1_	°	19
	Blade outlet angle	*β*_b2_	°	29.5
Volute	Inlet width	*b*_3_	mm	100
	Inlet diameter	*D*_3_	mm	365
	Tongue diameter	*D*_tongue_	mm	394
	Throat area	*A*_throat_	m^2^	0.0130
	Outlet diameter	*D*_d_	mm	200

**Table 2:** Impeller trimming schemes and parameter variation.

	Prototype	Scheme 1	Scheme 2	Scheme 3
*D*_2,2_ (mm)	365	354	343	332
Δ*D* (mm)	-	11	22	33
Δ*D*/*D*_2_	-	0.03	0.06	0.09
*A*_2_ (m^2^)	0.0499	0.0498	0.0496	0.0481
*β*_b2_ (°)	29.5	29.5	29.4	26.1

**Table 3:** Mesh information.

Location	Number of Elements (×10^4^)	Angle (°)	Aspect Ratio
Suction chamber	111.7	18.1	14
Impeller	242.7	25.9	149
Volute	190.8	20.2	49
Discharge pipe	39.8	55.1	78

**Table 4:** Boundary conditions.

Location	Boundary Type	Option
Inlet of suction chamber	Inlet	Total Pressure
Outlet of discharge pipe	Outlet	Mass Flow Rate
Entire physical surfaces	Wall	No Slip Wall

## Data Availability

The data presented in this study are available upon reasonable request from the corresponding author.

## Nomenclature

**Latin Letters**			
*A*_2_	Area of impeller outlet section	*Z*	Blade number
*D*_1_	Impeller inlet diameter	*b*_2_	Impeller outlet width
*A*_throat_	Volute throat area	*b*_3_	Volute inlet width
*D*_2_	Impeller outlet diameter	*n*	Rotating speed of impeller
*D*_3_	Volute inlet diameter	*p*_T1_	Total pressure at inlet of a component
*D*_d_	Volute outlet diameter	*p*_T2_	Total pressure at outlet of a component
*D*_s_	Suction inlet diameter	*s*	Specific entropy
*D*_tongue_	Volute tongue diameter	*u*_2_	Tangential velocity at blade trailing edge
*F*_x_	Radial force in x direction	*v*_2_	Absolute velocity at blade trailing edge
*F*_y_	Radial force in y direction	*v*_m2_	Meridional component of *v_2_*
*H*	Head	*v*_u2_	Circumferential component of *v_2_*
*P*	Power	*v*_x_	Component of absolute velocity in x direction
*P*_is_	Impeller input power	*v*_y_	Component of absolute velocity in y direction
*Q*	Flowrate	*v*_z_	Component of absolute velocity in z direction
*T*	Temperature	*w*_2_	Relative velocity at blade trailing edge
**Greek Letters**			
Δ*H*	Head drop of a component	*η*	Efficiency
Δ*H**	Loss coefficient	*μ*	Dynamic viscosity
*α*_2_	Flow angle at blade trailing edge	*ρ*	Density
*β*_b1_	Blade inlet angle	Φ*_D_*	Dissipation
*β*_b2_	Blade outlet angle	Φ_*D’*_	Turbulent dissipation
*ε*	Dissipation rate of turbulent kinetic energy	$\Phi_{\bar{D}}$	Viscous dissipation

## References

[1] Gülich J.F. (2014). Centrifugal Pumps.

[2] Sulzer Pumps (2010). Centrifugal Pump Handbook.

[3] Detert Oude Weme D.G.J., van der Schoot M.S., Kruyt N.P., van der Zijden E.J.J. (2018). Prediction of the effect of impeller trimming on the hydraulic performance of low specific-speed centrifugal Pumps. J. Fluids Eng..

[4] Gonzalez J., Parrondo J., Santolaria C., Blanco E. (2006). Steady and unsteady radial forces for a centrifugal pump with impeller to tongue gap variation. J. Fluids Eng..

[5] Šavar M., Kozmar H., Sutlović I. (2009). Improving centrifugal pump efficiency by impeller trimming. Desalination.

[6] Wang K., Zhang Z., Jiang L., Liu H., Li Y. (2017). Effects of impeller trim on performance of two-stage self-priming centrifugal pump. Adv. Mech. Eng..

[7] Yang S., Kong F., Jiang W., Qu X. (2012). Effects of impeller trimming influencing pump as turbine. Comput. Fluids.

[8] Sanjay V.J., Abhishek S., Karan H.M., Rajesh P. (2015). Effects of impeller diameter and rotational speed on performance of pump running in turbine mode. Energy Convers. Manag..

[9] Li C., Li X., Li P. (2014). Numerical investigation of impeller trimming effect on performance of an axial flow fan. Energy.

[10] Wang C., Shi W., Wang X., Jiang X., Yang Y., Li W., Zhou L. (2017). Optimal design of multistage centrifugal pump based on the combined energy loss model and computational fluid dynamics. Appl. Energy.

[11] Shi F., Yang J., Miao S., Wang X. (2019). Investigation on the power loss and radial force characteristics of pump as turbine under gas–liquid two-phase condition. Adv. Mech. Eng..

[12] Schmandt B., Herwig H. (2011). Internal flow losses: A fresh look at old concepts. J. Fluids Eng..

[13] Kock F., Herwig H. (2004). Local entropy production in turbulence shear flows: A high-Reynolds number model with wall functions. Int. J. Heat Mass Transf..

[14] Kock F., Herwig H. (2005). Entropy production calculation for turbulence shear flows and their implementation in cfd codes. Int. J. Heat Fluid Flow.

[15] Herwig H., Kock F. (2007). Direct and indirect methods of calculating entropy generation rates in turbulence convective heat transfer problems. Heat Mass Transf..

[16] Böhle M., Fleder A., Mohr M. Study of the Losses in Fluid Machinery with the Help of Entropy. Proceedings of the 16th International Symposium on Transport Phenomena and Dynamics of Rotating Machinery.

[17] Zhang F., Appiah D., Hong F., Zhang J., Yuan S., Adu-Poku K., Wei X. (2020). Energy loss evaluation in a side channel pump under different wrapping angles using entropy production method. Int. Commun. Heat Mass Transf..

[18] Gu Y., Pei J., Yuan S., Wang W., Zhang F., Wang P., Appiah D., Liu Y. (2019). Clocking effect of vaned diffuser on hydraulic performance of high-power pump by using the numerical flow loss visualization method. Energy.

[19] Li D., Wang H., Qin Y., Han L., Wei X., Qin D. (2017). Entropy production analysis of hysteresis characteristic of a pump-turbine model. Energy Convers. Manag..

[20] Osman M.K., Wang W., Yuan J., Zhao J., Wang Y., Liu J. (2019). Flow loss analysis of a two-stage axially split centrifugal pump with double inlet under different channel designs. Proc. Inst. Mech. Eng. Part C J. Mech. Eng. Sci..

[21] Guan H., Jiang W., Yang J., Wang Y., Zhao X., Wang J. (2020). Energy loss analysis of the double-suction centrifugal pump under different flow rates based on entropy production theory. Proc. Inst. Mech. Eng. Part C J. Mech. Eng. Sci..

[22] Brennen C.E. (1994). Hydrodynamics of Pumps.

[23] Hergt P., Krieger P. (1969). Radial forces in centrifugal pumps with guide vanes. Proc. Inst. Mech. Eng..

[24] Chu S., Dong R., Katz J. (1995). Relationship between unsteady flow, pressure fluctuations, and noise in a centrifugal pump-part B: Effects of blade-tongue interactions. J. Fluids Eng..

[25] Guo S., Okamoto H. (2003). An Experimental study on the fluid forces induced by rotor-stator interaction in a centrifugal pump. Int. J. Rotat. Mach..

[26] Tan M., Guo B., Liu H., Wu X., Wang K. (2015). Investigation of radial force and hydraulic performance in a centrifugal pump with different guide vane outlet angle. J. Vib..

[27] Hao Y., Tan L. (2018). Symmetrical and unsymmetrical tip clearances on cavitation performance and radial force of a mixed flow pump as turbine at pump mode. Renew. Energy.

[28] Jiang W., Li G., Liu P., Fu L. (2015). Numerical investigation of influence of the clocking effect on the unsteady pressure fluctuations and radial forces in the centrifugal pump with vaned diffuser. Int. Commun. Heat Mass Transf..

[29] Zou Z., Wang F., Yao Z., Tao R., Xiao R., Li H. (2016). Impeller radial force evolution in a large double-suction centrifugal pump during startup at the shut-off condition. Nucl. Eng. Des..

